# Vascular Complications in TAVR: Incidence, Clinical Impact, and Management

**DOI:** 10.3390/jcm10215046

**Published:** 2021-10-28

**Authors:** Markus Mach, Sercan Okutucu, Tillmann Kerbel, Aref Arjomand, Sefik Gorkem Fatihoglu, Paul Werner, Paul Simon, Martin Andreas

**Affiliations:** 1 Department of Cardiac Surgery, Medical University Vienna, 1090 Vienna, Austria; tillmann.kerbel@meduniwien.ac.at (T.K.); paul.werner@meduniwien.ac.at (P.W.); paul.simon@meduniwien.ac.at (P.S.); martin.andreas@meduniwien.ac.at (M.A.); 2 Department of Cardiology, Memorial Ankara Hospital, 06520 Ankara, Turkey; sercan.okutucl@memorial.com.tr; 3 Department of Cardiology, St. John of God Hospital, Geelong, VIC 3220, Australia; aref.arjomand@med.com; 4 Department of Cardiology, Iskenderun State Hospital, 31240 Hatay, Turkey; sgfatihoglu@gmail.com

**Keywords:** transfemoral, transcatheter, aortic valve, vascular, complications, TAVR, TAVI

## Abstract

Transcatheter aortic valve replacement (TAVR) has replaced surgical aortic valve replacement as the new gold standard in elderly patients with severe aortic valve stenosis. However, alongside this novel approach, new complications emerged that require swift diagnosis and adequate management. Vascular access marks the first step in a TAVR procedure. There are several possible access sites available for TAVR, including the transfemoral approach as well as transaxillary/subclavian, transcarotid, transapical, and transcaval. Most cases are primarily performed through a transfemoral approach, while other access routes are mainly conducted in patients not suitable for transfemoral TAVR. As vascular access is achieved primarily by large bore sheaths, vascular complications are one of the major concerns during TAVR. With rising numbers of TAVR being performed, the focus on prevention and successful management of vascular complications will be of paramount importance to lower morbidity and mortality of the procedures. Herein, we aimed to review the most common vascular complications associated with TAVR and summarize their diagnosis, management, and prevention of vascular complications in TAVR.

## 1. Introduction

Transcatheter aortic valve replacement (TAVR) has become the new standard of therapy for patients with severe aortic stenosis, and de facto replaced surgical aortic valve replacement (SAVR) when applicable [1,2]. Nevertheless, with the advent of this novel procedure, new complications emerged. Even though the first TAVR was performed via an antegrade transseptal approach, the transfemoral (TF) access is nowadays the most commonly used access strategy. It is applied in over 90% of all TAVR patients in most centers nowadays [1,2,3]. Vascular access is mainly achieved by puncturing the common femoral artery (CFA) and large bore sheaths that are advanced through retrograde access, and vascular complications are of particularly significant concern during TAVR. Alternative access strategies, via the apex or the ascending aorta as well as the transcarotid, transaxillary, or transcaval access, are performed in specific centers; however, they are not very widespread, primarily due to procedure-specific complexities. As the indication for TAVR is steadily moving towards lower-risk patients, an even stronger focus on the early diagnosis, adequate management, and prevention of these complications will be required for comparable results with SAVR. We hereby provide a broad overview of the most common vascular complications associated with TAVR, their effective management, and their prevention.

## 2. Materials and Methods

We performed a search of the PubMed database, Scopus, and the Web of Science using the keywords transcatheter aortic valve replacement (all fields) AND vascular (all fields) AND complications (all fields) (last update: 1 September 2021). There was no date or language restriction for our selection of publication. References of selected studies and all abstracts from cardiology congresses (American College of Cardiology, American Heart Association, European Society of Cardiology, PCR London Valves, and Transcatheter Cardiovascular Therapeutics) were searched for relevant data. Supplementary Figure S1 provides the PRISMA flowchart of studies included in this systematic review. Supplementary Table S1 provides an overview of vascular access complications and associated bleeding events in all studies analyzed in this review. The data were subdivided with respect to access routes, TAVR devices, and the application of VARC endpoint definitions. [1,2,4,5,6,7,8,9,10,11,12,13,14,15,16,17,18,19,20,21,22,23,24,25,26,27,28,29,30,31,32,33,34,35,36,37,38,39,40,41,42,43,44,45,46,47,48,49,50].

The manuscript aims to provide a concise and precise description of the experimental results, their interpretation, and the experimental conclusions that can be drawn.

## 3. Vascular Complications in TAVR

### 3.1. Incidence and Definition

The heterogenic group of intra-operative, as well as early postoperative, vascular complications are significantly associated with a higher rate of postinterventional morbidity and mortality, and it is alongside postinterventional pacemaker implantations as the most common type of complications after TAVR [47,51]. Especially in the early days of TAVR, vascular complications were relatively common, even though they varied widely around 2% and 30% due to unstandardized definitions of vascular complications [47,51]. Valve Academic Research Consortium (VARC) formulated standardized endpoint definitions for common adverse events after TAVR for better comparability between published data [52,53]. Three main subgroups were conceived as major vascular complications, minor vascular complications, and percutaneous closure device failure (Table 1) [53].

The PARTNER trial described vascular complications in almost a quarter of patients treated with TAVR, with a nearly even distribution of major (15.3%) and minor vascular complications (11.9%) using these definitions [47]. Genereux et al. reported a vascular complication rate of 11.9% in a meta-analysis with 3519 patients [54]. Current literature reporting outcomes, according to the updated standard VARC definitions, describe vascular complication rates ranging between 10% and 20% [51,55,56]. Comparing the relatively high vascular complication rates in the early days of TAVR, a significant decrease down to 4% and less can be observed in the recent literature [1,2,57,58,59]. However, vascular access complications are still quite common, with a major influence on adverse outcomes after TAVR [60]. Not only are they strongly correlated with increased hospitalization days, poorer quality of life outcomes, and 30-day and 1-year mortality, but also with bleeding complications, access site infections, and renal impairment leading to substantially increased procedural costs [51,61]. Observed 30-day mortality was significantly higher in patients with major vascular complications as opposed to those without vascular complications [51,61]. The PARTNER trial even demonstrated a four-fold increase in 30-day mortality in patients with major vascular access complications [47]. Notably, minor vascular complications have less impact on outcome and survival [62]. Endovascular experts or even vascular surgeons need to be firmly involved in heart team decisions and preoperative assessment to improve outcomes in TAVR patients and make this treatment applicable in young, low-risk patients. Vascular complications need to be diagnosed early and treated accordingly, but prevention will be pivotal for TAVR to be beneficial in younger patients with less surgical risk.

**Table 1:** Valve Academic Research Consortium-2 classification of vascular access site and access-related complications.

Complication	Definition
Major vascular complications	Any aortic dissection, aortic rupture, annulus rupture, left ventricle perforation, or new apical aneurysm/pseudoaneurysm; Access site or access-related vascular injury (dissection, stenosis, perforation, rupture, arterio-venous fistula, pseudoaneurysm, hematoma, irreversible nerve injury, compartment syndrome, percutaneous closure device failure) leading to death, life-threatening or major bleeding *, visceral ischemia, or neurological impairment; Distal embolization (non-cerebral) from a vascular source requiring surgery or resulting in amputation or irreversible end-organ damage; The use of unplanned endovascular or surgical intervention associated with death, major bleeding, visceral ischemia, or neurological impairment; Any new ipsilateral lower extremity ischemia documented by patient symptoms, physical exam, and/or decreased or absent blood flow on lower extremity angiogram; Surgery for access site-related nerve injury or permanent access site-related nerve injury.
Minor vascular complications	Access site or access-related vascular injury (dissection, stenosis, perforation, rupture, arterio-venous fistula, pseudoaneurysm, hematomas, percutaneous closure device failure) not leading to death, life-threatening or major bleeding *, visceral ischemia, or neurological impairment; Distal embolization treated with embolectomy and/or thrombectomy and not resulting in amputation or irreversible end-organ damage; Any unplanned endovascular stenting or unplanned surgical intervention not meeting the criteria for a major vascular complication; Vascular repair or the need for vascular repair (via surgery, ultrasound-guided compression, transcatheter embolization, or stent graft).
Percutaneous closure device failure	Failure of a closure device to achieve hemostasis at the arteriotomy site leading to alternative treatment (other than manual compression or adjunctive endovascular ballooning).

* Refers to VARC-2 bleeding definitions. Adapted and reproduced with permission from the copyright owner [63].

### 3.2. Risk Factors

Several procedural, as well as patient-related, factors contribute to the occurrence of vascular complications (Table 2). Female gender, peripheral vascular disease–especially in patients with a borderline femoral diameter and/or circumferential calcification patterns, a sheath-to-femoral-artery-ratio (SFAR) of less than 1.05 or a sheath diameter that exceeds the minimal femoral diameter, severe iliofemoral tortuosity patterns with an iliofemoral tortuosity score above 21.2, as well as operator experience and planned surgical cut-down are substantiated independent predictors of vascular complications [47,51,61,63,64,65,66,67]. High volume centers that can provide a sufficient learning curve to warrant adequate operator experience, meticulous patient selection as well as deliberate preoperative assessing measurements, and the use of low-profile sheaths (<19Fr) and valves of the newer generation substantially reduce the rate of such complications [61,68,69]. A further decline in vascular complication rates is expected due to the further development of vascular closure devices and smaller delivery systems.

### 3.3. Access Techniques

**Figure 1:**
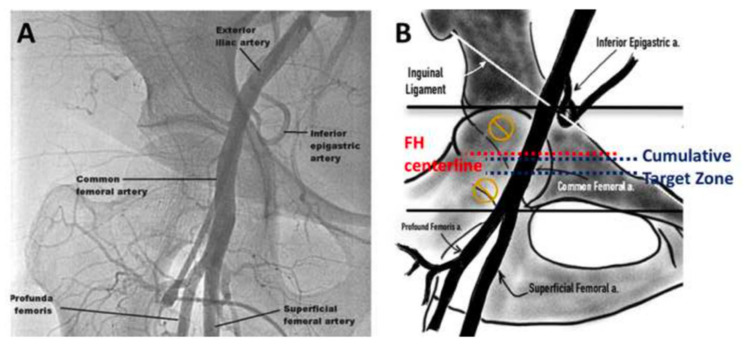
(**A**) Fluoroscopic and (**B**) schematic illustration of the ideal common femoral artery puncture site.

Diligent preprocedural assessment of the access vessels is crucial to select the best strategy for the patient and to keep vascular complications at a bare minimum. Contrast-enhanced multidetector computed tomography (MDCT) helps assess iliofemoral vessel diameters, calcification load and pattern distribution, tortuosity, and skin-to-artery distance. In the earlier days of TAVR, operators would mainly rely on traditional anatomical landmark guidance (TALG) for a vascular puncture, using the inguinal ligament and the zone of maximal femoral pulsation as a reference. Arterial puncture 2–3 cm caudally to this point in a 30–45° angle targets the CFA over the femoral head that serves as a firm counter bearing during manual compression for hemostasis. A low puncture, especially distally to the femoral bifurcation, should be avoided as it bears a higher risk for pseudoaneurysm or arteriovenous fistula formation, dissection, rupture, or thrombus formation [70]. A high puncture penetrating the external iliac artery or inferior epigastric artery will likewise impede achieving hemostasis and result in an eighteen-fold increase of risk for retroperitoneal bleedings [71]. Noteworthy, the sole reliance on anatomical features such as the skin crease will lead to a low puncture in 72% of patients and the zone of maximal femoral pulsation to a high puncture in 93% of patients [72]. Another approach to locate the optimal zone for arterial puncture is ultrasound-guided access. Therefore, a linear ultrasound probe is used to determine the height of femoral bifurcation and to exclude anterior wall calcification in the puncture zone. Identification of the artery is facilitated by the possibility of compression of the femoral vein. Real-time needle guidance reduces the risk of a posterior wall or sidewall puncture. Compared to fluoroscopy guidance, the vascular complications, the risk of venous puncture, and the number of attempts of successful vessel access were significantly reduced [73]. Although no study demonstrated a clear benefit of ultrasound or fluoroscopy-guided femoral access over TALG as a default strategy, it is potentially helpful in high-risk patients with profound vascular calcification or a marked skin-to-artery distance [73,74,75]. In such cases, a fluoroscopic target zone for safe CFA puncture can be defined in anterior-posterior projection between the centerline of the femoral head and a caudal 14mm margin avoiding both the femoral bifurcation and retroperitoneal vessels (Figure 1). Road mapping using digital subtraction crossover angiography via contralateral CFA access is another useful technique to mitigate the risk of access complications. Initial vascular access is usually performed using a micro-puncture needle and a 4–5F sheath to avoid large-bore needle trauma in case of an unsuccessful puncture and can later be exchanged over a standard guidewire.

Even though percutaneous closure devices offer good postoperative results and sheath sizes became notably smaller over the years, the surgical access offers a controlled and safe access, whose benefits might be overlooked in patients that are at risk for vascular complications [76,77,78,79]. While some studies demonstrated that surgical access is comparably safe and cost-effective, other studies indicated specific advantages of a percutaneous approach, especially with regard to access site infections [76,79,80,81,82]. The surgical cut-down is performed via a 30–40mm transversal incision starting right distally of the inguinal ligament and laterally from the femoral artery to preserve lymphatic integrity. The subcutaneous tissue is carefully dissected, and the femoral artery is prepared to place a purse-string suture or two U-sutures in a non-calcified spot on the CFA. Vascular access is then gained via direct puncture under direct visual control. After sheath removal, the sutures are tied. A femoral patch angioplasty or interposition grafting is mainly used when direct vascular closure cannot be achieved.

The subclavian access is the most frequent alternative access strategy to the transfemoral access and is usually performed from the left side for better valve alignment. Even though transaxillary TAVR is commonly performed over surgical cut-down, a fully percutaneous approach is feasible with puncturing the proximal third of the axillary artery. A minimum vessel diameter of 6mm is recommended, but in cases with prior coronary bypass grafting using the ipsilateral internal mammary artery, the vessel diameter should exceed 7 mm. Increased angulation at the aorto-subclavian junction favors kinking of the sheath or delivery system. Ectatic and severally calcified arteries should be avoided due to the increased risk for vascular complications that can be challenging to control [83].

Transcarotid (TC) TAVR has the main advantage of the short distance to the native aortic valve and the anatomically facilitated coaxial alignment; however, this access strategy is not widely performed due to its proximity to nerval structures and the respiratory tract, as well as its risk of stroke, even though experienced centers report similar stroke and vascular complication rates as via a transfemoral approach. TC-TAVR is usually performed under local anesthesia and cerebral near-infrared spectroscopy. A complete Circle of Willis is a prerequisite for the safety of this approach [84,85].

Depending on the anatomical position of the aorta, the transaortic access is performed either through a right anterior mini-thoracotomy in case of a right-sided ascending aorta or patent bypass grafts or through a median hemi-sternotomy in case of deep intrathoracic location or severe lung disease [86]. A minimum puncture to native aortic annulus distance of 8 mm for the Edwards Sapien 3 valve and 6mm for the Medtronic CoreValve is required [37,87]. Compared to the transapical approach, patients treated with transaortic TAVR are not at risk of ventricular scarring and subsequent development of apical pseudoaneurysm. Fiorina et al. demonstrated lower overall vascular complication rates predominantly driven by minor vascular complication transaortic TAVR patients compared to transaxillary TAVR patients [88]. However, direct comparisons to other access strategies are scarce, and observational studies and meta-analyses suggest similar mortality rates and vascular complications when compared to the transfemoral access [87,89,90,91,92].

The transapical access is performed over an anterolateral intercostal incision, puncturing the left ventricle at the apex cordis. Sufficient myocardial thickness and frailty must be considered, as apex closure can be cumbersome in patients with a tenuous free wall of the left ventricle. Hence, procedure-specific access complications such as left ventricular pseudoaneurysm formation and tamponade may occur. Even though access complications rates are low, it has been indicated that the transapical access is an independent predictor of higher postinterventional mortality [93,94,95].

If there is a lack of alternative access sites, transcaval access can be performed via femoral venous access. At the level of the inferior vena cava, an arteriovenous fistula is created by the application of electrocautery over a coronary guidewire. The transcatheter valve implantation is then carried out in a standard fashion, and the fistula is closed with an Amplatzer P.D.A. occlude or a similar device. There are limited outcome data, but with major vascular complications ranging between 11% and 28% and major or life-threatening bleeding rates between 13% and 28%, a significant learning curve must be considered as well as operator and center experience [96].

### 3.4. Guidewires, Catheters, and Sheaths

#### 3.4.1. Guidewires

Different guidewires need to be used during TAVR, but all usually come at a 0.0035″ diameter and an exchange length of 260 cm or more. They typically consist of a solid proximal core for adequate support and push-ability that is tapered towards the soft atraumatic tip, ensuring shape-ability and steerability. Some will have either hydrophilic coating, for increased lubricity and easier tracking to minimize vessel trauma, or hydrophobic coating, for a better tactile response. A wide range of wires are used and differ between institutions but most commonly include catheters of the Amplatz family (Boston Scientific, Marlborough, MA, USA), the Back-up Meier wire (Boston Scientific, Marlborough, MA, USA), the Lunderqvist Extra stiff (Cook Medical, Bloomington, IN, USA), and the Safari wire (Medtronic Inc). The Safari wire is pre-shaped with a distal exaggerated J curve, but other wires may need to be bent manually, with the rigid portion forming a part of the J curve. This will ensure good wire support and, more importantly, reduce the risk of vascular or left ventricular perforation. The flexural modulus describes the bending stiffness of wires in gigapascals (GPa) and was introduced by Harrison et al. to provide objective comparability between different products, as market terminology can be misleading [97]. High wire stiffness can be beneficial in cases with severe vascular tortuosity, but such wires require cautious handling.

#### 3.4.2. Catheters

A novel method to ensure safe passage of delivery systems in borderline-sized iliofemoral vessels is the use of intravascular lithotripsy. For this purpose, specifically designed catheter systems are used to disrupt intimal and medial calcifications through controlled microfractures and microdissections, thereby achieving an increase in vascular compliance. These catheter systems were evaluated in the DISRUPT-PAD I and II trials in patients with calcified femoropopliteal vascular lesions and demonstrated a surprisingly low incidence of vascular complications requiring intervention (1.7%) without displaying an increased rate of embolic debris in distal embolic filters [98,99].

Registry data of 42 patients with peripheral artery disease and otherwise prohibitive transfemoral access pathways showed that these intravascular lithotripsy catheters allowed safe transfemoral passage of TAVR delivery systems in more than 90% of all patients [100,101]. Within this small cohort, no iliofemoral dissections or perforations requiring intervention were reported, with only one patient (2.4%) demonstrating a pseudoaneurysm and another (2.4%) requiring endarterectomy [101].

#### 3.4.3. Sheaths

The insertion of larger sheaths in the CFA strongly correlates with higher vascular complication rates [102]. However, sheath size depends on the size and type of the implanted device. As such, a minimal femoral vessel diameter of 5.5 mm or a SFAR of less than 1.05 is recommended for transfemoral TAVR [51]. Due to ongoing developments of valve delivery systems, initial sheath sizes of 24 Fr and 26 Fr of the first generation of the Edwards Sapien valves and 25 Fr for the Medtronic CoreValve newer generation valves require 14Fr to 16Fr sheaths for balloon-expandable valves and 14Fr sheaths for self-expanding valves.

Three sheath designs need to be mentioned due to their innovative design. The eSheath (Edwards LifeSciences, Irvine, CA, USA) is a transiently expandable sheath with a length of 26 cm and has a 14Fr profile for the Sapien 3 valve. The sheath expands approximately 2 mm during valve passage and then returns to its original diameter. The sheathless EnVeo-R delivery system with its built-in 14Fr InLine sheath (Medtronic, Minneapolis, MN, United States) currently offers the lowest profile on the market. The SoloPath sheath (Terumo Medical Corporation, Irvine, CA, USA) is a 35 cm tapered balloon-expandable hydrophilic sheath. Its distal end is folded over a pre-mounted inflatable balloon dilatation catheter that can expand the sheath to 19Fr. Once the balloon is removed, the sheath will maintain its expanded shape and can return to its original size once the balloon is deflated. This design decreases vascular friction and trauma during sheath insertion in patients with borderline-sized femoral vessels. A single-arm study with 90 patients demonstrated the safety and efficacy of the Solopath sheath even in patients with a SFAR of greater than 1.05. Compared to patients with a SFAR of less than 1.05, no difference in procedural success and overall vascular or bleeding complications had been observed [103]. As low-profile sheaths (<19Fr) cause less vascular and bleeding complications [102], there is no evidence yet on the actual clinical benefit of expandable sheaths over fixed diameter. And even though smaller vessels can be tackled with such sheaths, valve passage through the sheath may not be feasible in all cases.

### 3.5. Hemostasis Methods

Transfemoral TAVR in its initial phase was predominantly performed via surgical cut-down, which is still a viable option in situations with morbidly obese patients, significant anterior calcification of the access vessel, alternative access sites (e.g., the subclavian access), and surgically experienced centers [80,104,105,106]. Even though reported outcomes demonstrate less vascular and bleeding complications, the reduction in sheath size and the increased use and evolution of pre-closure devices and techniques lead to the predominant use of a fully percutaneous access technique. Hemostasis after sheath removal is mainly achieved using suture-mediated closure devices such as the Prostar XL or Perclose ProGlide closure devices (Abbott Vascular Devices, Redwood City, CA, USA). A reduction in vascular access and bleeding complications had been demonstrated in several studies [81,82]. Even though these devices are indicated for closure of 10F (Prostar XL) and 8F (Perclose ProGlide) arteriotomy sites, if deployed before the initial sheath insertion—as in the “preclose” technique—generally, good hemostasis can be achieved [107,108]. The sutures are placed before large-bore sheaths are inserted, tied manually, and approximated with the help of knot pushers at the end of the procedure, once the sheath and the 0.0035″ guidewire are consecutively removed. A single Prostar XL device can close arteriotomies up to 19Fr using the pre-closure technique. If two devices are deployed at a 45° angle, sufficient closure of larger arteriotomy sites up to 24Fr can be achieved [109]. Similarly, such a “double preclose technique” can be applied using two Perclose ProGlide devices deployed at a 30–35° angle to create an interrupted x-figure suture for closing larger arteriotomy sites. This technique has proven to be effective and efficient with a low incidence of early and late closure site complications, as well as reduced hospital stay [104,110].

The MANTA VCD consists of a toggle placed within the vessel and a bovine collagen plug situated outside the artery. Both components are connected and pushed together, fixating each other on the internal and external vessel wall, leading to arteriotomy closure. Both device parts are completely resolvable within 6 months. The device got the C.E. mark for vascular closure for sheath sizes up to 22 French. Retrospective studies that compared MANTA to ProStarXL revealed comparable rates of vascular complications but significantly lower bleeding rates after MANTA application. A hybrid closure technique using both a suture (ProGlide) and a collagen (Angio-Seal) mediated closure device has been proposed with good results, having a high success rate (98%) and low vascular complications [111].

Even though some centers propagate the use of an ipsilateral double arterial access to reduce the use of contrast agents, most centers prefer an ancillary arterial at the contralateral side [112]. Both access strategies can be used to ensure vascular closure after sheath removal and control potential access site complications. However, contralateral access allows the application of the crossover balloon occlusion technique (CBOT). Therefore, an angioplasty balloon is inflated above the access site prior to sheath removal to temporarily reduce blood flow and, subsequently, blood pressure at the access site. This technique ensures safe and successful closure in patients undergoing TAVR with large bore-sheaths up to 24Fr. In unfavorable contralateral femoral anatomy, a transradial crossover approach can be used as a reasonable alternative [113].

### 3.6. Diagnosis and Management of Specific Vascular Complications

Most commonly, vascular access is gained over the CFA. Therefore, vessel dissection, rupture, access site hematoma, and the formation of pseudoaneurysms are all possible complications during or after TAVR. General measures such as blood volume substitution or medical resuscitation need to be promptly available. Other possible causes for a hemodynamic decline, such as coronary artery obstruction or valve function impairment, should be excluded immediately when suspected. In any case, both endovascular and surgical treatment must always be available to ensure a maximum safe environment for any patient treated with TAVR. Diagnostic crossover angiography to assess aortic or iliofemoral vascular complication after sheath removal is routinely advocated in most centers and is considered best clinical practice. This diagnostic maneuver is not only performed for early detection of vascular complication—arguably the most critical factor for optimal management—but also allows rapid vascular access through the placement of a crossover wire from the contralateral CFA. An overview of common vascular complications and their management is depicted in Table 3.

#### 3.6.1. Aortic Dissection or Rupture

**Figure 2:**
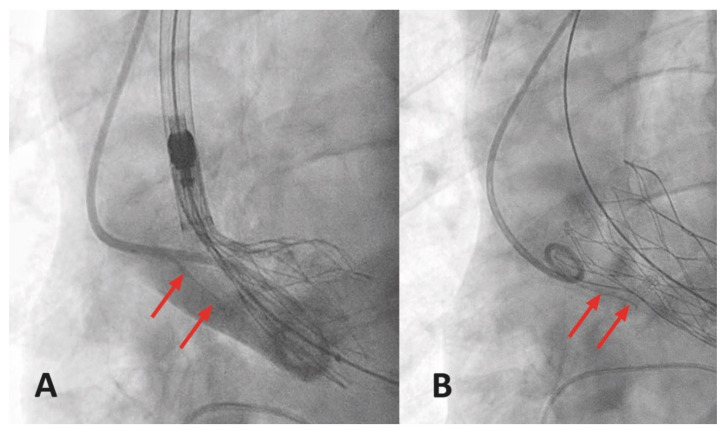
(**A**,**B**) Fluoroscopic evidence of dissection in the ascending aorta during THV deployment with red arrows indicating the dissection flap.

These complications occur quite rarely, but dissection and rupture of the aorta and especially the aortic annulus are catastrophic and immediately life-threatening complications. Even though the incidence with less than 2% is relatively low, the clinical impact is quite devastating, with mortality rates of up to 50% for aortic dissections [51,105,110,114]. The aortic root and ascending aorta can be injured by the expanding balloons, valves, or the delivery system itself. At the same time, catheters or guidewires can lead to injury of the intima leading to acute or subacute aortic dissection of Stanford type A. Typically, this mechanism occurs during valvuloplasty or valve implantation, especially in the case of device migration during the expansion phase (Figure 2). The dissection of the descending aorta without the involvement of the ascending aorta, as in a Stanford type B dissection, is an even rarer entity limited to single case reports and is mainly caused due to tip injury of the sheath at the time of delivery system introduction and advancement [115]. Patients may present with acute or subacute chest or abdominal pain or neurological or hemodynamic changes, depending on the location and limitations of the dissection. Most centers still rely on periprocedural transesophageal echo (TEE) during TAVR, even if transfemoral TAVR is increasingly performed under local anesthesia without TEE nowadays. Hence, periprocedural TEE and/or angiography may expedite such diagnosis if suspected early. Postprocedural CT-angiography (CTA) is commonly performed for affirmation. As Stanford type B dissections can be treated medically by limiting systolic arterial pressures to 100–110 mmHg and keeping M.A.P. over 70 mmHg, endovascular treatment with TEVAR may be necessary in some cases. Stanford type A dissections, on the other side, mandate immediate surgical treatment. Rupture of the aortic annulus that requires similar to aortic dissection surgical repair is mainly caused by oversizing of the valvuloplasty balloon or prosthesis in the presence of severe annular calcification extending in the muscular region of the LVOT (between right-to-left coronary cusp commissure and the left fibrous trigone), and especially in cases with an isolated bulky calcification of a single cusp [116]. A large multicenter study demonstrated that a higher annular calcification score was associated with landing zone rupture compared to patients with lower scores (181 ± 211 vs. 22 ± 37; *p* 0.001) [117]. Several other MDCT related parameters, including leaflet asymmetry defined as

$$\sqrt{\begin{array}{c} {}[\left(non\;coronary\;leaflet\;area-right\;coronary\;leaflet\;area\right)2\\ +\\ \left(right\;coronary\;leaflet\;area-left\;coronaryy\;leaflet\;area\right)2] \end{array}}$$

or the annular cover index defined as

$$\left[prosthesis\;nominal\;area-\frac{annular\;area}{prostehsis\;nomina\;area\;}\times \;100\right]$$

might add incremental predictive value during risk stratification in patients with a high risk for landing zone rupture. Valves that generate high radial forces, as well as post-dilatation in patients with THV valves implanted with >20% area oversizing, should be avoided in such cases [118].

Risky situations potentially occur when the valvuloplasty balloon recoils into the left ventricle during full expansion. Annular rupture occurs at rates around 1% and results in rapid development of hemopericardium and pericardial tamponade. Delayed clinical manifestations are rare but possible in slow-progressing or contained ruptures. MDCT-based assessment of aortic annulus dimension in conjunction with adapted sizing guidelines may reduce the incidence of severe oversizing [119,120]. The imminent importance of “heart team” on-site must be stressed again, as only immediate surgical intervention will control these life-threatening complications. Rupture of the descending or abdominal aorta can be managed by immediate balloon occlusion followed by either surgical or endovascular repair using covered stent-grafts.

#### 3.6.2. Iliofemoral Dissection or Rupture

The incidence of dissection of the CFA or iliac arteries ranges between 1.6% and 21.4% for a complete percutaneous transfemoral approach and between 2% and 7% for a surgical cut-down approach [51,61,68,107,121]. Dissections occur most likely in the external iliac artery during any phase between the initial vascular access and advancement of the delivery system, and they may not be observed until sheath withdrawal. Retrograde or contralateral antegrade control angiography prior to completion of TAVR usually reveals vascular access injuries, possibly leading to limb ischemia depending on the grade of vascular blood flow limitation, subsequent thrombus formation, and thromboembolic events. Postinterventional vascular Doppler, CTA, or angiography should be performed in case of acute onset of leg or back pain, clinical signs of ischemia, or hemodynamic deterioration. In case of compromised blood flow, angioplasty with prolonged balloon inflation alone may suffice for intima-media re-apposition. However, extensive dissection may require uncovered or even covered stent implantation or surgical treatment [68,107,121,122]. Asymptomatic small dissections without flow-limitation can be treated conservatively but need to be followed up closely.

Potentially fatal iliofemoral rupture is observed in 0.7% to up to 9.3% of TAVR procedures. Similar to iliofemoral dissections, lower incidence rates are displayed in more recent publications due to the introduction of low-profile sheath systems and sheathless delivery systems [68,107,121,122]. It is mainly detected after sheath withdrawal, as it usually seals the tear during the valve implantation [123,124]. Especially large bore sheath withdrawal from small, calcified vessels can be critical as it can lead to arterial avulsion [121,125]. The patient’s clinical status can deteriorate rapidly in case of extensive rupture and gradually over hours if small tears remain undetected. However, extraluminal contrast accumulation during final access site angiography is a clear indicator. Immediate sealing of the tear should be performed, either through the reintroduction of the sheath or contralateral balloon occlusion. Bleeding from smaller tears can resolve after a couple of minutes after occlusion and anticoagulation reversal. However, larger vessel injuries warrant immediate covered stent-graft implantation, surgical patch-repair, or interposition-grafting [123].

#### 3.6.3. Access Site Bleeding and Hematoma

Access site hematomas are relatively common, with reported incidences between 2.2 and 12.5%. Still, a steady decline can be observed due to increasing operator experience, use of low-profile sheaths, and advances in vascular closure techniques [51,104,126]. They appear either immediately reversal or gradually over hours to days of TAVR, despite manual compression and anticoagulation. They are generally benign and with spontaneous resolution over weeks, but they are associated with a prolonged hospital stay, secondary infection, need for blood transfusion, and increased mortality [127]. Diagnosed primarily during clinical examination, hematomas can be treated conservatively in most cases. Manual compression and anticoagulation reversal should be performed if oozing or active bleeding from the puncture site is apparent. Similar to small vascular tears, endovascular treatment involving prolonged balloon occlusion and self-expanding stent implantation can be indicated. Infrequently, large hematoma compressing nerval structures need to be evacuated surgically.

Retroperitoneal hemorrhage or hematoma can be due to aortic, iliac, inferior epigastric, or femoral injury and very often lead to nonspecific symptoms of groin, flank, or back pain with or without hemodynamic changes. If retroperitoneal hematomas are radiologically confirmed by CT or angiography, and with an overall incidence of up to 2.2%, most can be managed by transfusion of coagulation factors or red blood cell units [53,104,107]. In case of overt bleeding, coil embolization of small, ruptured vessels and covert stent-graft implantation or surgical repair in larger ruptures are indicated, depending on the size and location of the bleeding [53,104,107,126].

#### 3.6.4. Access Site Pseudoaneurysm

**Figure 3:**
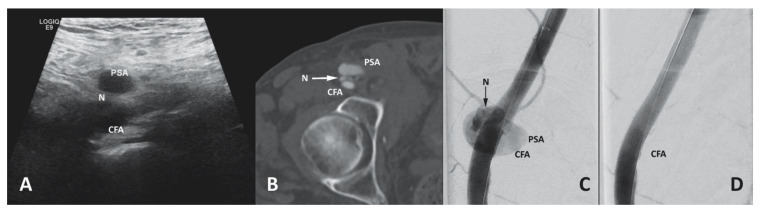
(**A**) Sonographic, (**B**) computer-tomographic, and (**C**) fluoroscopic evidence of pseudoaneurysm formation (PSA) with a short, broad neck (N) in the common femoral artery (CFA) after transfemoral THV implantation. (**D**) Pseudoaneurysm exclusion by endovascular implantation of a balloon-expandable covered stent graft (8 × 50 mm Gore^®^ Viabahn^®^).

Pseudoaneurysm (PSA) formation results in 2–6% of TAVR cases due to contained rupture with extravascular arterial bleeding into a pseudo-capsule [128]. Mostly diagnosed as a pulsatile mass in the groin during the clinical examination or Doppler ultrasound, a systolic bruit can be heard during auscultation. Several risk factors, such as advanced age, frailty, high B.M.I., current anticoagulation medication, the use of high-profile sheaths, high or low puncture, arterial and venous puncture, severe vascular calcification, and failed manual compression, can promote PSA formation [129]. Spontaneous PSA thrombosis is common in small pseudoaneurysm (3.0–3.5 cm) and patients without the necessity of anticoagulation [130,131]. A larger PSA leads to progressing discomfort, local infection, septic embolism, and rupture [131]. With a success rate of 97%, ultrasound-guided thrombin injection is favored over ultrasound-guided compression [132,133]. Incremental doses of 0.2–0.4 mL are injected until flow within the PSA ceases. Complication such as infection or thromboembolism is observed in less than 1% of cases [129]. Larger pseudoaneurysm unfavorable for thrombin injection require interventional or surgical occlusion (Figure 3). Similar to cut down for transfemoral TAVR, the CFA is approached, while sparing the PSA as much as possible, and clamped after heparin administration. Subsequently, the PSA is opened, and the puncture site is closed with either single sutures or a patch-plasty.

#### 3.6.5. Access Site Infection

The rate of access site infections ranges up to 6.3% in transfemoral cases and are more often reported after surgical cut-down than complete percutaneous access [51,126]. Partner 1A reported access site infections of 2% with no difference in sternal wound infection rates after SAVR [48]. Preventive measures include the administration of broad-spectrum antibiotics thorough prepping and draping prior to and after the procedure. Superficial infections respond well to local or systemic therapy if treated early. Infections involving periarterial tissue can lead to sepsis and substantially increase the risk of mortality [51,126]. Surgical debridement and V.A.C.© therapy (vacuum-assisted closure; Kinetic Concepts; KCI Medical, San Antonio, TX, USA) are the main therapeutic treatment options for deep wound infections.

#### 3.6.6. Closure Device Failure

**Figure 4:**
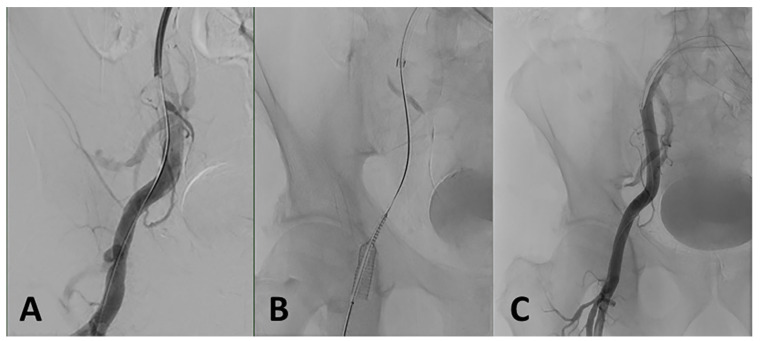
(**A**) Vascular closure device failure after TAVI with consecutive stenosis and bleeding of the right common femoral artery. (**B**) Endovascular treatment of the right common femoral artery with a self-expanding covered stent graft (9 × 50 mm Gore^®^ Viabahn^®^). (**C**) Control angiography shows unobstructed outflow without extravasation.

Closure device failure is described in 4.4 to 8.7% of cases and can cause arterial dissection, rupture, and vascular constriction or occlusion [51,104,107]. In case of insufficient hemostasis, treatment options are not different from access site oozing. Manual compression alone is sufficient in most cases. If limb ischemia is suspected, vascular Doppler, CTA, or angiography are appropriate to verify unrestricted blood flow. Angioplasty or stent implantation may be indicated in case of severe vascular stenosis or bleeding (Figure 4).

#### 3.6.7. Vascular Complications Associated with Non-Transfemoral Access

Access complications during TA-TAVR are rare but potentially fatal complications. Bleeding from the puncture site, or myocardial tears during access site closure, are the most frequent complications observed. As TAVR is usually performed in elderly patients, myocardial tissue can be rather soft and frail. Patients with a dilated left ventricle and a thin free wall are at particular risk. Apical hypokinesis can be observed during follow-up and is caused by myocardial scarring or close puncture to the left anterior descending artery, with closing suture limiting myocardial blood flow [93,134,135]. These complications can lead to ventricular aneurysm formation over time. Rib retraction and intercostal nerve damage can cause chronic chest pain at the access site and are less frequently observed when elastic soft tissue retractors are used instead of mechanical rib spreaders [134,135,136].

Vascular complications during or after transaxillary TAVR are limited to small series or single case reports. The pattern of vascular complications is similar to that seen with transfemoral access; however, achieving hemostasis with manual compression is rather difficult due to the lack of a supporting structure to buttress against during compression. Therefore, a low threshold towards endovascular stent implantation seems advisable, especially since closure device failure rates of 29.2% have been reported by Schäfer et al. Of note, the same study suggested the use of the ProGlide over the ProStar closure system since all closure device failures were related to the ProStar use. However, the outcome depends largely on the experience of the Heart Team [137].

As predominantly elderly patients are treated with transaortic TAVR, the ascending aorta may be soft and fragile, leading to tearing suture lines and a cumbersome arterial closure. Typical complications for the median hemi-sternotomy or anterior-lateral thoracotomy are deep sternal wound infections, mediastinitis, and right internal mammary artery injury. Very rarely, lacerations of the right ventricle during mini-thoracotomy and PSA formation of an intercostal artery after hemi-sternotomy has been described [76,138].

#### 3.6.8. Prevention Measures

With vascular access complications having a major impact on the outcome and mortality after TAVR, no effort must be spared to limit the risk of adverse events to a minimum. Thorough preoperative risk assessment involves detailed radiological and clinical preprocedural work-up. Multimodality imaging is pivotal for a tailor-made and patient-orientated approach warranting the safest access based on the individual vessel condition. CTA prior to the procedure is the foundation of an in-depth analysis of the patient’s anatomy and an integral part of risk stratification [139]. It is not only necessary for annular sizing and valve selection but also vital for access site analysis. Vessel diameters, calcification patterns, and tortuosity are integral to access site selection [67]. The International Society of Cardiovascular Computed Tomography (SCCT) has formulated recommendations in aortic valve and access site assessment prior to TAVR (Table 4) [140].

## 4. Conclusions

With TAVR now being an integral part of modern valvular interventions, the procedure has undergone an incredible evolution since first performed two decades ago. With the possibility to choose between many different access sites, ongoing technological advances in the valve design, sheath technology, and growing expertise, the rates of vascular access complications will continue their persistent decline. Even though TAVR is steadily gaining in simplicity and manual ease, we must not cease to focus on diligent vascular access and closure techniques, but, even more importantly, we must focus on preventive measures. Optimizing the strategies for vascular access in every individual patient, further miniaturizing sheath diameters and developing improved vascular closure devices will be mandatory to enhance the safety of transcatheter valve therapies.

## Figures and Tables

**Table 2:** Risk factors associated with vascular complications.

Risk Factors	
Non-modifiable	Gender (women men) Age (older younger) Obesity Peripheral vascular disease (SFAR 1.05, circumferential/ horseshoe calcification) Vascular tortuosity Blood dyscrasia
Modifiable	Puncture site (CFA SFA or EIA) Sheath size (LPS HPS) Puncture type (anterior wall only anterior + posterior wall; CFA only CFA + vein puncture) Anticoagulation regime

SFAR—sheath-to-femoral-artery-ratio; CFA—common femoral artery; SFA—superficial femoral artery; EIA—external iliac artery; LPS—low-profile sheath; HPS—high profile sheath. Adapted and reproduced with permission from the copyright owner [20].

**Table 3:** Management of vascular complications.

Location	Management
Aortic complications	
Aortic rupture	Open surgical repair Aortic occlusion balloon and cardiopulmonary bypass to stabilize
Aortic dissection	Surgical and endovascular repair Medical management
Iliofemoral complications	
Arterial perforation	Immediate reversal of anticoagulation Prolonged balloon angioplasty or, less commonly, covered stent-graft implantation from a contralateral or ipsilateral CFA access
Arterial dissection	Flow-limiting, prolonged balloon angioplasty or covered stent-graft implantation from a contralateral or ipsilateral CFA access
Arterial stenosis, thrombosis, and occlusion	Thrombectomy or balloon angioplasty
Pseudoaneurysm	Size <3.0–3.5 cm: observation Size >3.0–3.5 cm or expanding: thrombin injection
Hematoma	Conservative, manual compression, prolonged balloon angioplasty from contralateral CFA access

Adapted and reproduced with permission from the copyright owner [66].

**Table 4:** SSCT expert consensus on CT evaluation before TAVR.

**Recommendations for assessment of access route by CT before TAVR**
CT imaging should be performed for vascular access assessment (pelvic arteries and aorta) when not contraindicated. CT examinations should be performed with iodinated contrast medium. Manual multiplanar reformation or semi-automated centerline reconstruction should be used to achieve cross-sectional visualization to measure vessel dimensions. From these reconstructed images, the minimal luminal diameter along the course of the vascular access should be determined. Qualitative assessment of vascular tortuosity should be performed. Qualitative assessment of vascular calcification should be performed. Consideration of varied thresholds of vessel size (sheath/femoral artery ratio) should be contemplated, depending on the presence and extent of vascular calcification. The left ventricle should be evaluated for the presence of thrombus and, if a transapical access route is planned, for geometry and position of the apex.
**Recommendations for assessment of the aorta before TAVR**
The entire aorta should be imaged and evaluated, unless a transapical access is planned. Severe elongation and kinking of the aorta, dissection, and obstructions caused by thrombus or other material should be reported.

Adapted and reproduced with permission from the copyright owner [140].

## Data Availability

The datasets for this study will be available from the corresponding author upon reasonable request.

## Supplementary Materials

The following are available online at https://www.mdpi.com/article/10.3390/jcm10215046/s1, Figure S1: Preferred Reporting Items for Systematic Reviews and Met-Analysis (PRISMA)-flowchart, Table S1: Vascular access and access-site related bleeding complications reported for TAVR.
