# Supplementary material for: Vascular Complications in TAVR: Incidence, Clinical Impact, and Management
Source: J Clin Med. 2021 Oct 28;10(21):5046. doi: 10.3390/jcm10215046 (PMC8584339; doi:10.3390/jcm10215046)
Supplement: Supplementary file 1 [file jcm-10-05046-s001.zip › jcm-1377599-supplementary.pdf]

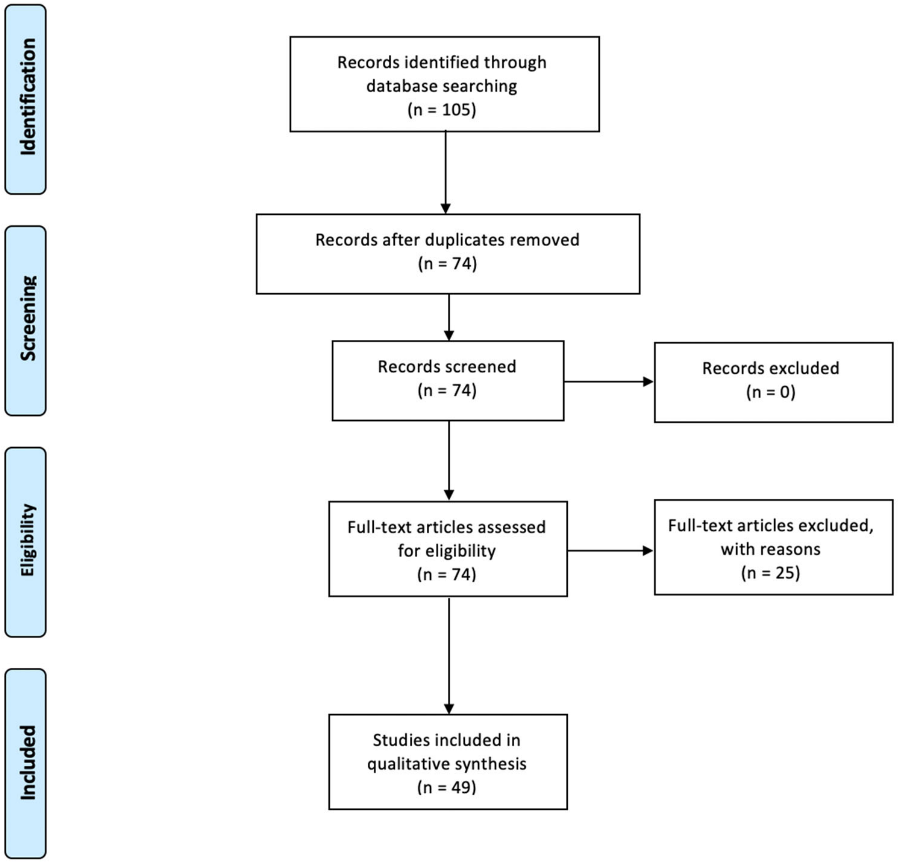

Supplementary Figure S1: Preferred Reporting Items for Systematic Reviews and Met-Analysis (PRISMA)-flowchart

Supplementarty Table S1: Vascular access and access-site related bleeding complications reported for TAVR

Author (year)	Trial name	Country	Centre n	Population n	Investigation period	Study design	Device	Access						Vascular complication			Access-site related bleeding				VARC (Y/N)
								TF n (%)	TA n (%)	S-CL n (%)	T-CA n (%)	DAO n (%)	T-CV n (%)	All n (%)	Major n (%)	Minor n (%)	All n (%)	L-Th n (%)	Major n (%)	Minor n (%)	
van Wiechen et. al (2021)	MASH	N, FR	2	206	2018–2020	RCT	S3, ER, EP, Ac. Neo, Lotus	206 (100)	0 (0)	0 (0)	0 (0)	0 (0)	0 (0)	14 (6.8)	2 (1)	12 (5.8)	n/a	15 (7.3)	n/a	n/a	Y
Akodad et. al (2020)	DIRECTAVI	FR	1	236	2016–2018	RCT	S3	n/a	n/a	n/a	n/a	n/a	n/a	n/a	7 (3)	n/a	n/a	n/a	7 (3)	n/a	Y
Yong et al. (2020)	SOLACE-AU	AUS	11	199	2012–2016	Prosp.	S XT	199 (100)	0 (0)	0 (0)	0 (0)	0 (0)	0 (0)	0 (0)	12 (7)	n/a	n/a	7 (3.5)	n/a	n/a	Y
Makkar et al. (2020)	PORTICO IDE	US, AUS	52	750	2014–2017	RCT	S (Gen 1), S XT, S3, CV (Gen 1), ER, EP, Portico	690 (92)	n/a	n/a	n/a	n/a	n/a	n/a	59 (7.9)	n/a	n/a	36 (4.8)	n/a	n/a	Y
Waksman et al. (2020)	LRT	US	6	61	2016–2019	Prosp.	S3, ER, EP	61 (100)	0 (0)	0 (0)	0 (0)	0 (0)	0 (0)	n/a	1 (1.6)	n/a	n/a	1 (1.6)	n/a	n/a	Y
Lanz et al. (2019)	n/a	MN	20	731	2017–2019	RCT	Ac. Neo, S3	731 (100)	0 (0)	0 (0)	0 (0)	0 (0)	0 (0)	123 (16.8)	49 (6.7)	75 (10.3)	174 (23.8)	23 (3.1)	74 (10.1)	81 (11.1)	Y
Khan et al. (2019)	BASILICA	US	4	30	2018–2018	Prosp.	S3, ER, EP	23 (76.7)	0 (0)	1 (3.3)	0 (0)	0 (0)	6 (20)	n/a	6 (20)	n/a	n/a	2 (6.7)	n/a	n/a	Y
Toutouzas et al. (2019)	DIRECT	GR, ISR	4	171	2015–2018	RCT	CV (Gen 1), ER, EP	165 (96.5)	0 (0)	6 (3.5)	0 (0)	0 (0)	0 (0)	30 (17.5)	8 (4.7)	22 (12.9)	54 (31.6)	3 (1.8)	21 (12.3)	30 (17.5)	Y
Mack et al. (2019)	PARTNER III	MN	71	496	2016–2017	RCT	S3	496 (100)	0 (0)	0 (0)	0 (0)	0 (0)	0 (0)	34 (6.9)	11 (2.2)	23 (4.6)	43 (8.7)	6 (1.2)	13 (2.6)	24 (4.8)	Y
Popma et al. (2019)	EVOLUT low risk	MN	86	734	2016–2018	RCT	CV (Gen 1), ER, EP	727 (99)	0 (0)	4 (0.5)	0 (0)	3 (0.4)	0 (0)	n/a	28 (3.8)	n/a	n/a	18 (2.45)	n/a	n/a	Y
Barbanti et al. (2019)	FAST-TAVI	MN	10	499	2015–2017	Prosp.	S3	499 (100)	0 (0)	0 (0)	0 (0)	0 (0)	0 (0)	n/a	9 (1.8)	n/a	n/a	n/a	n/a	n/a	Y
Beve et al. (2019)	n/a	FR	1	191	2009–2017	n/a	S (Gen 1), S XT, S3, CV (Gen 1), ER	0 (0)	104 (54.5)	73 (38.2)	14 (7.3)	0 (0)	0 (0)	2 (1.1)	0 (0)	2 (1.1)	21 (11)	0 (0)	15 (7.9)	6 (3.1)	n/a
Feldman et al. (2018)	REPRISE III	MN	55	912	2014–2015	RCT	CV (Gen 1), ER, Lotus	912 (100)	0 (0)	0 (0)	0 (0)	0 (0)	0 (0)	n/a	58 (6.4)	n/a	110 (12.1)	63 (6.9)	47 (5.2)	0 (0)	Y
Yamawaki et al. (2018)	n/a	J	14	1458	2013–2016	Prosp.	S XT, S3, CV (Gen 1)	1190 (81.6)	255 (17.5)	6 (0.4)	0 (0)	7 (0.48)	0 (0)	n/a	79 (5.4)	n/a	n/a	79 (5.4)	n/a	n/a	Y
Denegri et al. (2018)	n/a	CH	1	73	2015–2016	Prosp.	Portico	69 (94.5)	0 (0)	4 (5.5)	0 (0)	0 (09	0 (0)	10 (13.7)	3 (4.1)	7 (9.6)	15 (20.6)	6 (8.2)	0 (0)	9 (12.3)	Y
Seeger et al. (2018)	n/a	GER	1	400	2010–2015	Prosp.	CV (Gen1), S XT, S3, Lotus	400 (100)	0 (0)	0 (0)	0 (0)	0 (0)	0 (0)	n/a	29 (7.3)	n/a	n/a	n/a	n/a	n/a	Y
Hengstenberg et al. (2017)	BRAVO III	MN	31	802	2012–2015	RCT	n/a	802 (100)	0 (0)	0 (0)	0 (0)	0 (0)	0 (0)	n/a	75 (9.4)	n/a	n/a	n/a	n/a	n/A	Y
Reardon et al. (2017)	SURTAVI intermediate risk	MN	87	864	2012–2016	RCT	CV (Gen1), ER	n/a	n/a	n/a	n/a	n/a	n/a	n/a	52 (6)	n/a	n/a	105 (12.2)	n/a	n/a	Y

Popma et al. (2017)	EVOLUT R U.S	US	23	241	2014–2015	Prosp.	ER	214 (88.8)	0 (0)	16 (6.6)	0 (0)	9 (3.7)	0 (0)	35 (14.5)	18 (7.5)	19 (7.9)	25 (14.5)	17 (7.1)	12 (5)	7 (2.9)	Y
Takimoto et al. (2016)	n/a	J	6	299	2013–2015	Prosp.	S XT	200 (66.9)	99 (33.1)	0 (0)	0 (0)	0 (0)	0 (0)	n/a	5 (1.7)	n/a	n/a	7 (2.3))	n/a	n/a	Y
Lansky et al. (2016)	Neuro-TAVI	US	5	44	2014–2015	Prosp.	S (Gen 1), S XT, CV (Gen 1)	43 (97.7)	1 (2.3)	0 (0)	0 (0)	0 (0)	0 (0)	n/a	5 (11.4)	n/a	n/a	3 (6.8)	n/a	n/a	Y
Seeger et al. (2016)	n/a	GER	1	558	2007–2014	Retrosp.	S XT, S3, CV (Gen 1), Lotus	558 (100)	0 (0)	0 (0)	0 (0)	0 (0)	0 (0)	98 (17.6)	37 (6.6)	61 (10.9)	133 (23.8)	20 (3.6)	36 (6.5)	77 (13.8)	Y
Manoharan et al. (2016)	n/a	UK, GER	6	102	n/a	Prosp.	Portico	102 (100)	0 (0)	0 (0)	0 (0)	0 (0)	0 (0)	10 (9.8)	6 (5.9)	4 (3.9)	37 (36.27)	4 (3.9)	13 (12.8)	22 (21.6)	Y
Wöhrle J et al. (2016)	n/a	GER	1	110	2014–2015	Prosp.	Lotus	110 (100)	0 (0)	0 (0)	0 (0)	0 (0)	0 (0)	n/a	5 (4.6)	n/a	n/a	3 (2.7)	n/a	n/a	Y
Leon et. al (2016)	PARTNER IIa	MN	57	1011	2011–2013	RCT	S XT	775 (76.7)	236 (23.3)	0 (0)	0 (0)	0 (0)	0 (0)	n/a	80 (7.9)	n/a	n/a	105 (10.4)	n/a	n/a	Y
Wöhrle et al. (2016)	n/a	GER	1	235	2014–2015	Prosp.	S3	235 (100)	0 (0)	0 (0)	0 (0)	0 (0)	0 (0)	n/a	7 (3)	n/a	n/a	n/a	n/a	n/a	Y
Webb et al. (2015)	PARTNER IIb	US	28	560	2011–2012	RCT	S (Gen 1), S XT	560 (100)	0 (0)	0 (0)	0 (0)	0 (0)	0 (0)	105 (18.8)	69 (12.3)	35 (6.3)	195 (34.8)	56 (10)	99 (17.7)	48 (8.6)	Y
Binder et al. (2015)	n/a	CH	n/a	598	2011–2014	Prosp.	S XT, S3	598 (100)	0 (0)	0 (0)	0 (0)	0 (0)	0 (0)	83 (13.9)	46 (7.7)	36 (6)	80 (13.4)	30 (5)	43 (7.2)	7 (1.2)	n/a
Manoharan et al. (2015)	n/a	MN	6	60	2013–2014	Prosp.	ER	59 (98.3)	0 (0)	0 (0)	0 (0)	1 (1.7)	0 (0)	7 (11.7)	5 (8.3)	2 (3.3)	n/a	3 (5)	n/a	3 (5)	Y
Abramowitz et al. (2015)	n/a	US	1	734	2012–2014	n/a	S (Gen 1), S XT, S3	499 (68)	38 (5.2)	4 (0.55)	0 (0)	57 (7.8)	0 (0)	87 (11.9)	23 (3.1)	64 (8.7)	n/a	4 (0.6)	30 (4.1)	n/a	Y
Bosmans et al. (2015)	ADVANCE	MN	44	1015	2010–2011	Prosp.	CV (Gen 1)	n/a	n/a	n/a	n/a	n/a	n/a	n/a	111 (10.9)	n/a	321 (31.6)	n/a	110(10.8)	n/a	Y
Wendt et al. (2015)	n/a	GER	1	54	2013–2015	Prosp.	S3	0 (0)	41 (75.9)	0 (0)	0 (0)	13 (24.1)	0 (0)	0 (0)	0 (0)	0 (0)	n/a	n/a	7 (13)	0 (0)	Y
Castellant et al. (2015)	FRANCE 2 Registry	FR, MCO	34	3751	2010–2011	Registry	S (Gen 1), CV (Gen 1)	2247 (59.9)	673 (17.9)	211 (5.6)	0 (0)	n/a	n/a	343 (9.1)	174 (4.6)	169 (4.5)	518 (13.8)	74 (2)	155 (4.1)	289 (7.7)	Y
Baumbach et al. (2015)	DEFLECT I	MN	6	37	n/a	Prosp.	S (Gen 1), CV (Gen 1)	n/a	n/a	n/a	n/a	n/a	n/a	n/a	3 (8.1)	n/a	n/a	3 (8.1)	2 (5.4)	2 (5.4)	Y
Fearon et al. (2014)	PARTNER NRCA	MN	27	1438	2009–2012	Prosp.	S (Gen 1)	1438 (100)	0 (0)	0 (0)	0 (0)	0 (0)	0 (0)	275 (19.1)	147 (10.2)	128 (8.9)	n/a	n/a	132 (9.2)	40 (2.8)	N
Reardon et al. (2014)	CV Extreme Risk	US	41	150	2011–2012	Prosp.	CV (Gen 1)	0 (0)	0 (0)	70 (46.7)	0 (0)	80 (53.3)	0 (0)	n/a	14 (9.3)	n/a	n/a	43 (28.6)	60 (40)	n/a	N
Watanabe et al. (2015)	n/a	J, FR	4	301	2010–2012	Prosp.	S XT	159 (52.8)	67 (22.3)	0 (0)	0 (0)	75 (24.9)	0 (0)	n/a	25 (8.3)	n/a	n/a	n/a	n/a	n/a	Y
Stabil et al. (2014)	SAT-TAVI	IT	1	120	2010–2011	RCT	S XT	120 (100)	0 (0)	0 (0)	0 (0)	0 (0)	0 (0)	11 (9.2)	3 (2.5)	8 (6.7)	15 (12.5)	7 (5.8)	4 (3.3)	4 (3.3)	Y
Abdel-Wahab et al. (2014)	CHOICE	GER	5	241	2012–2013	RCT	S XT, CV (Gen 1),	241 (100)	0 (0)	0 (0)	0 (0)	0 (0)	0 (0)	32 (13.3)	25 (10.4)	7 (2.9)	60 (24.9)	24 (10)	40 (16.6)	20 (8.3)	N

Adams et al. (2014)	CV high risk Pivotal	US	45	390	2011–2012	RCT	CV (Gen 1)	390 (100)	0 (0)	0 (0)	0 (0)	0 (0)	0 (0)	n/a	23 (5.9)	n/a	n/a	53 (13.6)	109 (28)	n/a	n/a
Sawa et al.(2014)	n/a	J	MC	55	2011–2012	Prosp.	CV (Gen 1)	44 (80)	0 (0)	5 (9.1)	0 (0)	6 (10.9)	0 (0)	n/a	6 (10.9)	n/a	n/a	7 (12.7)	n/a	n/a	Y
Popma et al. (2014)	CV Extreme Risk	US	41	489	2011–2012	Prosp	CV (Gen 1)	489 (100)	0 (0)	0 (0)	0 (0)	0 (0)	0 (0)	n/a	40 (8.2)	n/a	n/a	62 (12.7)	121 (24.7)	n/a	Y
Seco et al. (2014)	n/a	AUS	1	32	2009–2013	Retrosp.	S (Gen 1)	0 (0)	32 (100)	0 (0)	0 (0)	0 (0)	0 (0)	1 (3.1)	1 (3.1)	0 (0)	14 (43.8)	2 (6.3)	5 (15.6)	7 (21.))	Y
Czerwińska-Jelonkie-wicz et al. (2014)	n/a	POL	1	83	1009–2011	Retrosp.	S (Gen 1), S XT, CV (Gen 1)	59 (71.1)	16 (19.3)	8 (9.6)	0 (0)	0 (0)	0 (0)	44 (53)	17 (20.5)	27 (32.5)	n/a	n/a	n/a	n/a	Y
Holper et al. (2014)	n/a	US	1	30	2011–2011	RCT	S (Gen 1)	30 (100)	0 (0)	0 (0)	0 (0)	0 (0)	0 (0)	8 (26.7)	5 (16.7)	3 (10)	n/a	n/a	n/a	n/a	Y
Généreux et al. (2012)	PARTNER Ia + Ib	MN	MC	419	n/a	RCT	S (Gen 1)	419 (100)	0 (0)	0 (0)	0 (0)	0 (0)	0 (0)	114 (27.2)	64 (15.3)	50 (11.9)	95 (22.7)	n/a	63 (15)	32 (7.6)	N
Smith et al. (2011)	PARTNER Ia	MN	25	348	2007–2009	RCT	S (Gen 1)	244 (70.1)	104 (29.9)	0 (0)	0 (0)	0 (0)	0 (0)	59 (17)	38 (10.9)	21 (6)	n/a	n/a	32 (9.2)	n/a	N
Leon et. al (2010)	PARTNER	MN	21	179	2007–2009	RCT	S (Gen 1)	179 (100)	0 (0)	0 (0)	0 (0)	0 (0)	0 (0)	45 (25.1)	29 (16.2)	26 (14.5)	n/a	n/a	30 (16.8)	n/a	N
Bleiziffer et. al (2009)	n/a	GER	1	152	2007–2008	Retrosp.	S (Gen 1), CV (Gen 1)	121 (79.6)	26 (17.1)	3 (2)	0 (0)	2 (1.3)	0 (0)	25 (16.5.)	n/a	n/a	n/a		n/a	n/a	N

Abbreviations: Ac. Neo—Acurate Neo; CV—CoreValve; ER—CoreValve Evolut R; EP—CoreValve Evolut Pro; DAO—direct aortic; Gen—Generation; MN—multinational; Prosp—Prospective study; RCT—Randomized Controlled Trial; Retrosp.—Retrospective study; S—Sapien Generation 1; S XT—Sapien XT, S3—Sapien 3; S-CL—subclavian; TA—transapical; T-CA—trans-carotid; T-CV—trans-caval; TF—transfemoral; VARC—Valve Academic Research Consortium .
